# Multi-omics uncovers nutrient stress-driven interactions in a synthetic *Prymnesium parvum* holobiont, with vitamin B_12_-limitation revealing mutualism

**DOI:** 10.1093/ismeco/ycag142

**Published:** 2026-05-22

**Authors:** Lou Patron, Florian Petrilli, Anaëlle Bout, Marinna Gaudin, Léna Gouhier, Clarisse Hubert, Damien Réveillon, Samuel Chaffron, Enora Briand, Matthieu Garnier

**Affiliations:** IFREMER, PHYTOX, GENALG, Nantes 44300, France; IFREMER, PHYTOX, GENALG, Nantes 44300, France; IFREMER, PHYTOX, METALG, Nantes 44300, France; IFREMER, PHYTOX, GENALG, Nantes 44300, France; IFREMER, PHYTOX, PHYSALG, Nantes 44300, France; IFREMER, PHYTOX, METALG, Nantes 44300, France; IFREMER, PHYTOX, METALG, Nantes 44300, France; CNRS, LS2N, Nantes Université, UMR 6004, Nantes 44000, France; IFREMER, PHYTOX, GENALG, Nantes 44300, France; IFREMER, PHYTOX, GENALG, Nantes 44300, France

**Keywords:** microalgae-bacterial interactions, metabarcoding, metatranscriptomics, metabolomics, toxins, synthetic community

## Abstract

Microalgal-bacterial interactions are central to nutrient cycling and ecosystem functioning in marine habitats, yet the mechanisms structuring these associations under defined nutrient constraints remain poorly resolved. Using a synthetic 15-member bacterial community (SynCom) and controlled nitrogen (N), phosphorus (P), and vitamin B_12_-limitation, we investigated how nutrient scarcity shapes the physiology, metabolism, and transcriptional activity of the harmful alga *Prymnesium parvum* and its associated microbiota. Under N- and P-limitation, the SynCom had minimal impact on algal growth despite nutrient-dependent shifts in toxin production and metabolite profiles. In contrast, B_12_-limitation triggered a strong mutualistic interaction in which the SynCom enabled a three-fold increase in algal biomass and drove restructuring of intracellular and extracellular metabolomes, including the accumulation of ectoine and membrane-associated lipids and the depletion of thiamine-like and stress-associated metabolites. Metabarcoding revealed stable community composition but enrichment of B_12_-producing taxa under B_12_-limited conditions, while metatranscriptomics uncovered functional divergence among SynCom members. Bacteria displayed contrasting response strategies, including nutrient-responsive specialists and transcriptionally flexible generalists maintaining gene expression across all nutrient regimes. B_12_-producers (*Marinovum algicola, Roseobacter* sp., *Halomonas* sp.) upregulated cobalamin biosynthesis exclusively under B_12_-limitation, whereas several B_12_-auxotrophic taxa induced B_12_-transport and B_12_-requiring enzymes, indicating active vitamin exchange within the holobiont. *P. parvum* displayed transcriptional programs specific to each nutrient limitation, with B_12_-limited co-cultures shifting toward growth-associated gene expression despite constitutive expression of the B_12_-dependent *metH* gene. These results demonstrate that vitamin auxotrophy acts as a key metabolic lever reorganizing holobiont function, driving reciprocal benefits and reprogramming algal-bacterial metabolism in our synthetic system.

## Introduction

Microalgae, as major constituents of phytoplankton, play an essential role in marine ecosystems by regulating key biogeochemical processes such as carbon sequestration, nutrient cycling, and oxygen production [1]. In natural environments, microalgae are associated with complex microbial consortia within the phycosphere, the microscale environment immediately surrounding algal cells [2] where symbiotic exchanges of metabolites can occur [3]. This web of interactions constitutes the algal holobiont, a functional ecological unit in which the algal host and its associated microorganisms interact metabolically and physiologically [4]. Reciprocal exchanges within the holobiont can range from mutualism to parasitism, collectively influencing the growth, nutrient acquisition, secondary metabolism, and community dynamics. Such chemically mediated interactions are increasingly recognized as key regulators of harmful algal bloom dynamics [5]. Because these interactions are highly sensitive to nutrient availability [6], examining how nutrient limitations affect holobiont organization and function is critical to understanding microalgal physiology and microbial assemblage dynamics.

At a global scale, nutrient availability largely controls marine primary production, including macronutrients such as nitrogen (N) and phosphorus (P), and micronutrients such as trace metals or organic growth factors (e.g. vitamin B_1_, B_7_, and B_12_) [7]. At a cellular level, nutrient limitation alters algal metabolism and growth. N-limitation promotes the biosynthesis and accumulation of carbon-rich storage compounds, notably lipids and carbohydrates [8–11] while P-limitation impairs photosynthesis, triggers extensive lipid remodeling [12–15], and enhances alkaline phosphatases secretion [16]. Vitamin B_12_-limitation constrains growth in auxotrophic microalgae, primarily by blocking methionine metabolism [17]. Whereas macronutrient limitation generally leads to reduced growth rates coupled with extensive metabolic reprogramming, vitamin B_12_ deprivation in auxotrophic microalgae can result in severe growth arrest, underscoring their reliance on bacterial cobalamin supply [18–21]. Yet, despite this well-established auxotrophy, the mechanisms governing how microalgae access, acquire, and compete for B_12_ with their associated microbial communities remain poorly understood [22].

Nutrient limitation also affects the bacterial partners within the phycosphere. Bacteria adjust their metabolism to scavenge limiting N and P, notably through high-affinity transporters and enzymes, like alkaline phosphatases [23, 24], and substitution of membrane phospholipids with non-P lipids [25, 26].

Indirectly, nutrient limitation alters algal physiology and exudation patterns, modifying the composition and lability of dissolved organic matter, thereby potentially selecting specific heterotrophic populations [27, 28]. Under B_12_-limitation, algal growth is constrained, and bacteria capable of supplying the vitamin might gain a selective advantage, thus reshaping community composition [18, 29]. Overall, these interconnected responses illustrate that nutrient stress shapes both algal and bacterial physiology, making a holobiont-level approach essential to understand microalgal ecology from the micro-scale to ecosystem-scale.

Among the thousands of isolated microalgal species, less than 200 are known to produce toxins [30] and can form harmful algal blooms with major ecological and economic consequences [31]. In these species, nutrient availability influences not only growth and bloom dynamics but also toxin biosynthesis [32], making them highly informative models to connect environmental pressures, physiological and metabolic responses, and microbial interactions.

In this study, we use *Prymnesium parvum* as a model to explore how nutrient limitation shapes microalgal-bacterial interactions. *P. parvum* is a mixotrophic haptophyte with global distribution [33]. It is auxotrophic for vitamin B_12_ [34] and produces potent ichthyotoxins, the prymnesins, which have the potential to disrupt gill function in fish and have ecological and economic consequences worldwide [35–37]. Although prymnesin production increases under N and P stress [38], it remains unclear whether this response, whose underlying mechanisms remain to be elucidated, differs across nutrient limitations, and how bacterial communities influence growth and toxin production [39, 40].

Controlled holobiont models, including synthetic microbial communities (SynComs), offer a tractable alternative to study complex microbial interactions. SynCom-based studies have demonstrated that harmful algae can selectively enrich particular bacterial taxa [39], and that bacteria can protect algae from pathogens [41]. However, most SynCom studies focus on taxonomic or functional responses alone, while multi-omics approaches combining both algal and bacterial partners remain limited.

Here, we extend SynCom-based approaches to investigate *P. parvum*-bacteria interactions under N-, P-, and vitamin B_12_-limitation. By combining a multifaceted methodological approach including metabarcoding, metatranscriptomics, and metabolomics, we investigated both the taxonomic and functional responses of algal and bacterial partners across nutrient limitation regimes. This holistic framework aimed to evaluate how different nutrient limitations shape *P. parvum* holobiont assemblage and modulate biotic interactions, as well as the physiology, metabolism, and functional activity of both algal and bacterial partners. Specifically, we tested whether nutrient-specific bacterial assemblages and functional profiles drive distinct transcriptional and metabolic responses within the holobiont, and how these changes influence algal growth and toxin production. Through this approach, we aim to advance towards a mechanistic understanding of nutrient-dependent processes that underpin both algal ecology and biogeochemical functioning at larger scales.

## Material and methods

### Co-culture experimental design

Fifteen bacterial strains were selected for their ecological relevance and functional diversity, including vitamin B_12_ production and diazotrophy (Tables 1 and S1), and assembled into the synthetic community (SynCom) as described previously [39]. All the strains were inoculated in equal proportional representation within the SynCom. *P. parvum* (CCAP 646/6) was acclimated for two weeks in L1 medium [42] under N- (N-lim; N adjusted to 62 μM), P- (P-lim; P adjusted to 2.2 μM), or vitamin B_12_-limited (B_12_-lim; B_12_ adjusted to 7.4 × 10^−6^ μM) conditions.

*P. parvum* cultures were inoculated at 1 × 10^5^ cells ml^−1^. In co-cultures treatments, the defined SynCom was added at a total density of 1 × 10^5^ cells ml^−1^. Cultures were grown in photobioreactors under continuous light (100 μmol photons m^−2^ s^−1^), at 20°C, and pH 8.2, in a phenotyping bench as previously described [43]. Four biological replicates with SynCom and three without were monitored for 14 days and ecophysiological parameters were measured as described previously [39].

**Table 1:** Composition of the synthetic microbial community (SynCom) used in this study.

Phylum/Class	Species	Particular functional trait
**Bacteroidota**	*Cyclobacterium* sp.	
	*Maribacter* sp.	
	*Muricauda* sp.	
**Alphaproteobacteria**	*Marinovum algicola*	B_12_ producer
	*Marivita cryptomonadis*	
	*Roseobacter* sp.	B_12_ producer
	*Thalassospira* sp.	B_12_ producer
**Gammaproteobacteria**	*Alteromonas* sp. 002	
	*Alteromonas* sp. 005	
	*Halomonas alkaliphila*	B_12_ producer
	*Halomonas* sp.	B_12_ producer
	*Marinobacter* sp.	
	*Vibrio aestuarianus*	
	*Vibrio diazotrophicus*	Diazotroph
	*Pseudomonas stutzeri*	Diazotroph

The table lists the 15 bacterial strains, their taxonomic affiliation (phylum or class), and a functional trait of interest (vitamin B_12_ production or diazotrophy).

### Growth and nutrient monitoring

Algal and bacterial abundances were monitored daily respectively using a Multisizer 4 Coulter Counter (Beckman Coulter, Indiana, USA), and by flow cytometry (MACSQuant Analyzer 10, Miltenyi Biotec, Germany) after SYBR Green I staining (1%).

Nutrient concentrations (N, P, C) were measured on Days 0, 3, 7, 9, and 14. For each photobioreactor, 15 ml of culture was filtered through pre-combusted GF/C filters, filtrates were used to quantify dissolved inorganic N and P, and filters were kept for particulate organic carbon (POC) and N (PON) analyses. Dissolved N and P were measured on a Seal AutoAnalyzer (SEAL Analytical, Germany) using classical colorimetric methods [44] adapted for marine samples [45]. POC and PON were quantified by elemental analyzer (Flash 2000, Thermo Fisher Scientific, USA).

To confirm the limiting nutrient at the end of the experiment, cultures were individually supplemented with N, P, or vitamin B_12_ and growth recovery was monitored.

### 16S metabarcoding

16S-metabarcoding was performed as previously described [39] on the Syncom at Day 0 and in the cultures at Days 3, 7, 10, and 14 of the experiment. Briefly, samples were sequentially filtered through polycarbonate membranes of 3 μm and 0.2 μm pore size to separate *P. parvum*-associated bacteria (PA) from free-living bacteria (FL). DNA was extracted (NucleoSpin Plant II, Macherey-Nagel, Germany), amplified (16S, V3-V4 region, primers 301F (CCTAYGGGRBGCASCAG)/805R (GGACTACNNGGGTATCTAAT)) and sequenced on Illumina MiSeq 2x250bp (ADNid, France). Sequences were processed using the SAMBA pipeline (https://gitlab.ifremer.fr/bioinfo/workflows/samba; v4.0.0), with taxonomic assignment of ASVs performed using SILVA (v138.1) as reference. Statistical analyses were conducted in R (v4.4.2) as described in section Statistical analyses. The sequencing dataset was deposited in the European Nucleotide Archive (ENA) under the project number PRJEB106332 and available here: https://doi.org/10.12770/cc2788fb-2915-411a-82d6-f832884835c7.

### Metabolomic and toxin analyses

On Day 8, intracellular and extracellular metabolites were analyzed, including targeted prymnesin (PRM1 + PRM2) quantification and untargeted metabolomics. Cells and supernatants were separated by centrifugation (50 ml, 3000 × g, 5 min at 4°C). Pellets were extracted twice with 1 ml of methanol (> 99.9% purity, CHROMASOLV™, Honeywell, USA), in an ultrasonic bath (15 min at 25 kHz). The two extracts were pooled, a subsample was ultrafiltered (0.2 μm, Nanosep MF, Pall, USA) and transferred into a glass vial while supernatants were processed by solid-phase extraction (SPE) using a Bond Elut C8 cartridge (500 mg, 10 ml, LRC, Agilent, USA), as previously described [46]. Blank extraction samples (empty tube for the intra-, and L1 medium for the extracellular fraction) were prepared as samples. A mixture of all samples was prepared for the qualitative control (QC) samples (intra- and extracellular extracts pooled separately). The injection order was randomized and QCs were injected every five samples.

Data were acquired on a UHPLC system (1290 Infinity II, Agilent, USA) coupled to a high-resolution quadrupole time-of-flight mass spectrometer (Q-Tof 6550 iFunnel, Agilent, USA), and processed as described previously for PRM [46] and for metabolomics [39].

Three successive filtering steps based on signal-to-noise ratio with blanks (S/N > 10), variability among pools (CV >30%) and autocorrelation were applied using in-house scripts on R [39]. The raw data matrices obtained were provided in Tables S4 and S5.

For the features significantly affected, MS/MS spectra were acquired by targeted MS/MS, at three collision energies (10, 30 and 50 eV). Spectra were processed using GNPS [44], SIRIUS v6.1.1 [45], and Flash entropy search [46] with the database retrieved as previously described [47]. Annotation levels ranged from 5 (exact mass) to 2 (probable structure by comparison with literature/library spectrum) [48]. The full dataset of targeted PRM1 and PRM2 quantification for intracellular and extracellular samples is provided in Table S2. All metabolomic data (full scan, MS/MS) are available at https://doi.org/10.12770/22b6f4c5-efc1-446c-903d-cc2729cac9ad as well as levels of annotation in Table S3.

### Transcriptomic analysis

Metatranscriptomic analyses were performed with cultures harvested on Day 14. Cultures were centrifuged to obtain algal and bacterial-enriched fractions (50 ml, 500 × g then 4000 × g (10 min each)). Total RNA from the bacterial enriched fractions was extracted following a TRIzol-chloroform protocol. Briefly, cell pellets were resuspended in 1 volume of TRIzol reagent and incubated for 10 min at 0°C. Chloroform was added (0.2 volumes), samples were vortexed for 5 min at room temperature, and centrifuged at 12 000 × g for 15 min at 4°C. The aqueous phase was transferred to a new tube and RNA was precipitated with 0.8 volume of 100% isopropanol, followed by incubation for 2 hours at −20°C. RNA pellets were washed with 70% ethanol, air-dried, and resuspended in nuclease-free ultrapure water. RNA concentration was quantified following the AccuBlue® Broad Range RNA Quantitation Kit protocol (Biotium, USA) on a Qubit fluorometer (Thermo Fisher Scientific, USA). Genomic DNA was removed using DNase I (EURx, Poland) following the manufacturer’s instructions, and rRNA depletion was performed using the QIAseq FastSelect kit (Qiagen, Netherlands) according to the manufacturer’s protocol. RNA integrity was assessed with a Bioanalyzer (Agilent, USA). Three samples per condition were sequenced (Illumina, Integragen, France), yielding 37–44 M paired-end reads per sample. Reads were trimmed (Cutadapt v4.1 [49]), residual rRNA removed (Bowtie2 v2.5.4 [50]) and mapped (HISAT2 v2.2.1 [51]) to the *P. parvum* genome (https://doi.org/10.12770/90ecb6f5-e981-4192-a653-72576bfd50c1) and to the 15 bacterial genomes sequenced in this study (Table S1). Details about read distribution as well as the taxonomic composition of SynCom mRNA reads are available in Figs. S1 and S2, respectively. Annotations of bacterial genomes were generated by merging the outputs of Bakta v1.11.4 [52], Prokka (v1.14.5) [53], and MaGe (v3.17.5) [54], all run with default parameters to reduce pipeline-specific biases. Count matrices for *P. parvum* and bacterial community genes were analyzed using DESeq2 (v1.46.0) [55] in R (https://www.r-project.org/) (v4.4.2). The sequencing dataset was deposited in the European Nucleotide Archive (ENA) under the project number PRJEB106332 and available here: https://doi.org/10.12770/cc2788fb-2915-411a-82d6-f832884835c7.

### Statistical analyses

Differences in PRM contents, and cellular C/N ratios were assessed by a one-way ANOVA with Tukey’s HSD. Metabolomic data were log-transformed and Pareto-scaled prior statistical analyses using Metaboanalyst 6.0 (www.metaboanalyst.ca). For intracellular data, the matrices were additionally normalized by cell number before analysis (Table S6). The fourth B_12_-lim replicate *with SynCom* was identified as an outlier and excluded from all subsequent analyses. Principal component analysis (PCA), volcano plots (|log₂FC| > 2, padj <0.05) and heatmaps were generated on the processed datasets.

Amplicon sequencing data were normalized using cumulative-sum scaling in SAMBA. Community variation across nutrient conditions, fractions (PA vs FL), and time points was explored using both PCA and non-metric multidimensional scaling (NMDS) based on Bray-Curtis dissimilarities. Differences in community composition were statistically tested using PERMANOVA (adonis2, vegan package), which provided R^2^ and p-values quantifying the proportion of variance explained by each factor.

For *P. parvum* transcriptomes, ribosomal protein genes were removed, raw counts normalized (median-of-ratios), and Differentially Expressed Genes (DEGs) identified using Wald statistics as implemented in DESeq2 (|log₂FC| > 3, padj <0.05). Log₂-transformed counts were clustered using pheatmap.

For bacterial metatranscriptomes, high sparsity prevented direct DESeq2 application. A three-step gene filter was applied: detection in ≥2 replicates, mean expression >10 within a condition, and both criteria satisfied in ≥2 nutrient conditions; genes meeting the first two criteria in only one condition were labelled “ON” and defined as being exclusively expressed in that condition. Filtered genes were analyzed with DESeq2 using the same statistical thresholds as for *P. parvum*. Functional enrichment of bacterial DEGs was performed with clusterProfiler.

All statistics and figures (except for metabolomic PCA and metabarcoding barplots) are available on gitlab (https://gitlab.ifremer.fr/lp7b2b6/horus/).

## Results

### Growth monitoring

**Figure 1:**
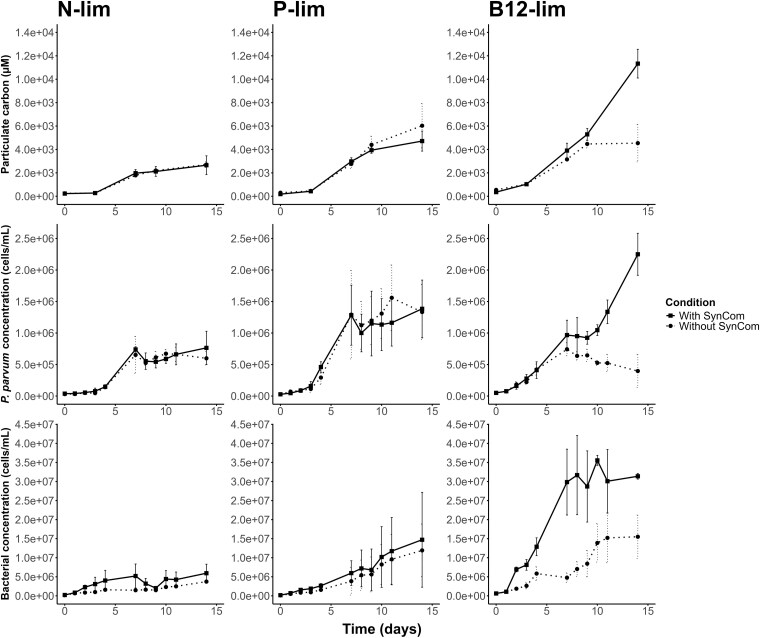
Growth dynamics of *P. parvum* and bacteria under nitrogen (N-lim), phosphorus (P-lim), and vitamin B_12_ (B_12_-lim) limitation, with and without a synthetic bacterial community (SynCom). Time-series of particulate organic carbon in μM (top row), algal cell concentration in cells/ml (middle row), and bacterial cell concentration in cells/ml (bottom row) in *P. parvum* cultures grown under nitrogen (left column), phosphorus (middle column), and vitamin B_12_ (right column) limitation. For each condition, cultures were supplemented (with SynCom, solid lines) or not (without SynCom, dashed lines) with a defined synthetic bacterial community at the beginning of the experiment.

*Prymnesium parvum* was grown under N-, P-, and B_12_-lim (n = 4 with SynCom; n = 3 without SynCom). Cultures entered stationary phase at Day 7 in all conditions. However, in B_12_-lim + SynCom, growth resumed on Day 10, ultimately reaching particulate carbon concentrations and cell densities 3.5 times higher at Day 14. (Fig. 1). In B_12_-lim, algal cells sizes were significantly larger without SynCom between Days 7 and 10 (Fig. S3; one-way ANOVA, F_1, 26_ = 42.82, *P* = 6 × 10^−7^; Tukey’s HSD). This SynCom-dependent recovery suggests active provision of vitamin B_12_ by bacterial partners. Nutrient-spiking assays at Day 15 confirmed that N and P limitations were successfully established. In the case of B_12_, the limitation remained effective in cultures without the SynCom, with growth resuming only upon exogenous B_12_ addition, whereas in B_12_-lim + SynCom, bacterial B_12_ production alleviated the limitation (Figs. S4–S6). Bacterial dynamics were similar under N- and P-lim ± SynCom and B_12_-lim without SynCom. Bacterial abundances were consistently higher with SynCom (1.6-fold in N-lim; 1.3-fold in P-lim). SynCom’s growth was significantly enhanced in B_12_-lim, from the third day onwards ultimately reaching levels ~5-fold higher than under N-lim and 2-fold higher than under P-lim.

### Toxin analysis

**Figure 2:**
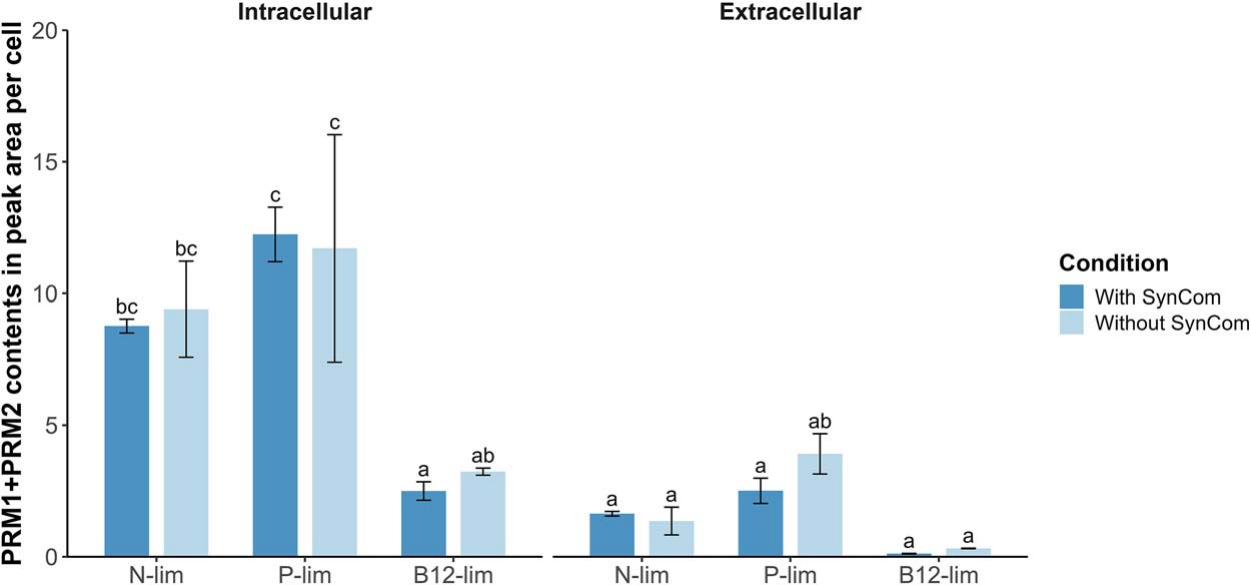
Intra- and extracellular prymnesin contents under different nutrient-limited conditions. Barplots show prymnesin levels (sum of PRM1 and 2, expressed as peak area per cell, mean ± SD) measured in N-lim, P-lim, and B_12_-lim *P. parvum* cultures, either with or without the presence of the SynCom. Bars are grouped by prymnesin fraction (intracellular vs extracellular) for each nutrient-limited condition. Measurements were performed at Day 8 of the experiment.

Intracellular and extracellular PRM1 and PRM2 were quantified on Day 8, when *P. parvum* was in a comparable physiological state across treatments, based on growth curves. At this time, most cultures were entering the early stationary phase, while B_12_-lim + SynCom cultures were at the beginning of the plateau phase prior to the recovery of growth, which was not yet anticipated. PRM content was strongly influenced by nutrient limitation (three-way ANOVA: Limitation F_2, 30_ = 25.31, *P* = 3.63 × 10^−7^), with P-lim and N-lim cultures showing higher levels than B_12_-lim. In contrast, the presence of the SynCom did not significantly affect PRM content (Condition F_1, 30_ = 0.26, *P* = .61), neither globally nor in interaction with nutrient limitation or fraction type. Extracellular PRM were on average approximately threefold lower than intracellular levels, but followed the same relative pattern across limitations (Fig. 2).

### Metabolomic analysis

**Figure 3:**
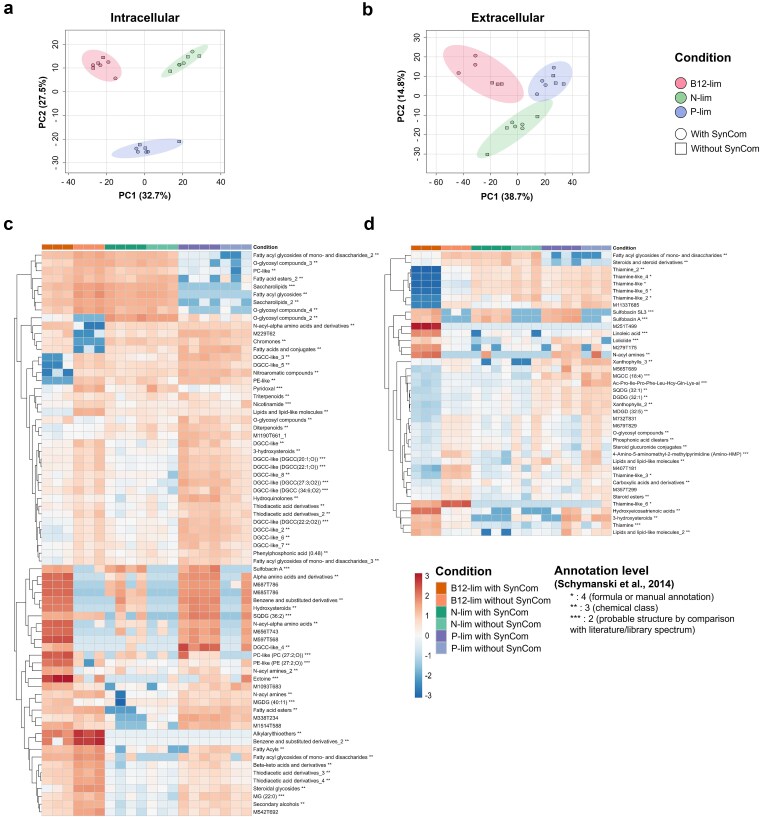
Multivariate and feature-level intra- and extracellular metabolomic responses to nutrient-limitation and SynCom presence. **(a)** PCA of intracellular metabolites measured in *P. Parvum* cultures under N-, P- and B_12_-lim, grown with or without the SynCom. Each point represents an individual sample, colored according to the nutrient-limitation condition and shaped according to SynCom presence or absence. **(b)** PCA of extracellular metabolites from the same cultures and conditions as in (a). **(c)** Heatmap of intracellular metabolic features significantly affected by the SynCom under B_12_-lim. Relative abundances of these features are shown across all nutrient-limited conditions (N-, P-, and B_12_-lim), with and without the SynCom, after normalization. Feature annotations indicate identification confidence levels following the classification proposed by [48]. **(d)** Heatmap of extracellular metabolic features significantly affected by the SynCom under B_12_-lim, displayed across all nutrient-limitation and SynCom treatment conditions as in (c).

Metabolomic profiles revealed strong nutrient-dependent responses, as both intra- and extracellular fractions clearly clustered by nutrient conditions (Fig. 3a and b), with a distinct clustering associated with SynCom presence in the extracellular fraction under B_12_-lim in the PCA (Fig. 3b). This structuring, driven by nutrient limitation, indicates distinct stress-specific metabolic responses, with B_12_-lim modulated by the SynCom. Comparisons of ± SynCom cultures highlighted 112 significantly modulated features including 72 intracellular and 40 extracellular in B_12_-lim, six extracellular in N-lim and seven extracellular in P-lim (Table S3). Among those systematically affected by the presence of the SynCom, we identified several extracellular and intracellular putative sulfobacins as well as intracellular α-amino-acid and benzene derivatives, hydroxysteroids and several unknown features. Among those affected by the presence of the SynCom in B_12_-lim only, with SynCom specific features, distinct intracellular and extracellular metabolic changes were observed. Intracellularly, putative compounds such as diacylglyceryl carboxy-hydroxymethylcholine (DGCC)-like lipids, phosphatidylethanolamine (PE)-like lipids and one nitroaromatic compound were depleted, while ectoine and multiple phosphatidylcholine (PC)- and PE-like lipids accumulated. Extracellularly, putative thiamine-like compounds, glycerolipids, steroids and glycosylated metabolites were reduced, whereas conjugated fatty acids, thiamine and the unknown feature M251T499 were strongly enriched (Fig. 3c and d).

### Metabarcoding analysis

The composition of the SynCom was followed over time and across nutrient limitations using 16S rRNA gene amplicon sequencing on both FL and PA fractions. A global NMDS analysis indicated that time was the main source of explained variation (R^2^ = 0.21, *P* < .001), with fraction type (FL vs. PA; R^2^ = 0.14, *P* < .001) and nutrient limitation (R^2^ = 0.12, *P* < .001) also contributing significantly to community structuring.

**Figure 4:**
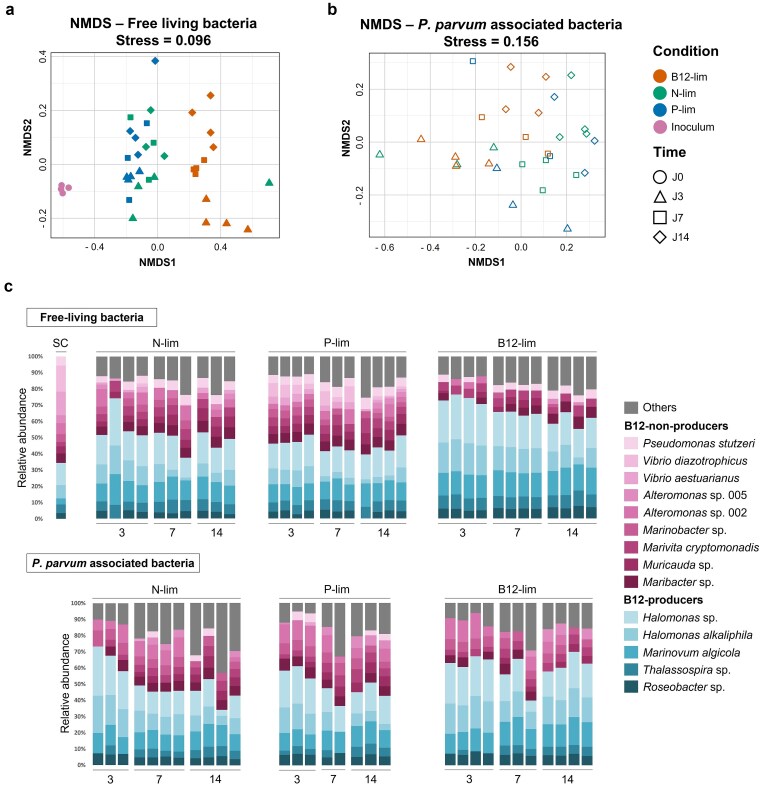
Dynamics of free-living and *P. Parvum*-associated bacterial communities under nutrient limitation. **(a)** NMDS ordination of bray-Curtis dissimilarities based on 16S rRNA metabarcoding data from free-living (FL) bacterial communities, obtained by filtration through 3 μm and 0.2 μm filters. Points are colored according to nutrient-limitation condition (N-, P-, or B_12_-lim) and shaped according to sampling time (0, 3, 7, 14 days). **(b)** NMDS ordination of *P. parvum*-associated (PA) bacterial communities presented as in (a). **(c)** Relative abundance of SynCom bacterial taxa in FL (top) and PA (bottom) fractions across nutrient-limitation conditions and time points. Barplots show the 14 SynCom strains detected by 16S rRNA metabarcoding (with *Cyclobacterium qasimii* excluded as it was not detected), while all non-SynCom taxa are grouped as “others” (grey). The “SC” bar represents the initial SynCom composition at T0 prior to inoculation. Taxa are grouped according to their vitamin B_12_ production capability, distinguishing B_12_-producing and B_12_-non-producing strains.

When considering each fraction separately, nutrient limitation primarily structured the FL community (R^2^ = 0.20, *P* < .001), with time exerting a secondary influence (R^2^ = 0.14, *P* < .001). In contrast, temporal variation dominated in the PA fraction (R^2^ = 0.45, *P* < .001), while nutrient limitation contributed more modestly (R^2^ = 0.11, *P* = .019; Fig. 4a and b).

Across all conditions, 14 of the 15 SynCom strains were consistently detected. Due to its low abundance (below the rarity threshold), *Cyclobacterium qasimii* was removed during the SAMBA bioinformatic decontamination pipeline and excluded from subsequent analyses. Only ASVs corresponding to SynCom members were retained, with all other sequences grouped as “Others” (Fig. 4c).

Several SynCom taxa exhibited fraction- and condition-specific patterns. *Pseudomonas stutzeri* was enriched in the FL fraction across all conditions, whereas *Vibrio* strains occurred exclusively in the FL fraction under P-lim. Both *Alteromonas* strains, were specifically enriched in the PA fraction under B_12_- and P-lim. Finally, B_12_-producing bacteria (*Marinovum algicola, Thalassospira* sp., *Roseobacter* sp., *Halomonas alkaliphila*, and *Halomonas* sp.) were enriched in B_12_-lim cultures, which together reached 60% ± 4% relative abundance at Day 14, compared to 46% ± 5% under N- and P-lim. Despite these condition-dependent shifts, overall community structure remained relatively consistent across nutrient regimes. Variation partitioning indicated that time explained the largest proportion of variance (R^2^ = 21%), whereas nutrient condition and fraction accounted for 12% and 14%, respectively. This suggests that the SynCom maintained a conserved core structure while allowing targeted enrichment of B_12_ producers under vitamin limitation.

### Transcriptomic analysis

To investigate molecular mechanisms underlying differences in community structure and potential interactions between *P. parvum* and the SynCom under nutrient limitations, we analyzed Day 14 transcriptomes for N- and P-lim with the SynCom, and for B_12_-lim with and without the SynCom. This time point was selected because clear differences in growth dynamics were observed in the growth curves by Day 14. Although this represents a single time-point snapshot, it captures a stage where the systems have diverged substantially, providing valuable insights into the transcriptomic activities of both *P. parvum* and the bacterial consortium and into how nutrient availability shapes their interactions.

#### *Prymnesium parvum* transcriptional responses

**Figure 5:**
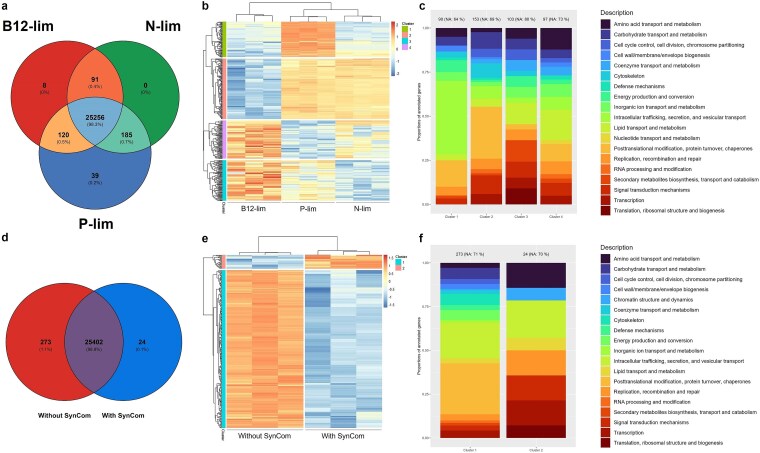
Transcriptional responses of *P. parvum* to nutrient limitation and SynCom presence. **(a)** Venn diagram showing the sets of *P. parvum* genes expressed under N-, P-, and B_12_-lim in the presence of the SynCom. **(b)** Heatmap of genes significantly differentially expressed across nutrient-limited conditions in the presence of the SynCom, clustered according to shared expression patterns. **(c)** Functional overview of transcriptional clusters identified in (b). Barplots summarize the main functional categories represented in each of the four clusters; bar colors correspond to functional groups. Numbers above bars indicate the total number of genes per cluster, with “NA: X” denoting the number of unannotated genes. **(d)** Venn diagram showing the sets of *P. parvum* genes expressed under B_12_-lim in the presence or absence of the SynCom. **(e)** Heatmap of genes significantly differentially expressed between B_12_-lim cultures with and without the SynCom, clustered based on shared expression profiles. **(f)** Functional overview of transcriptional clusters identified in (e). Barplots summarize the main functional categories represented in each of the two clusters; bar colors and annotations are as in (c).

Analysis of *P. parvum* mRNA on Day 14 revealed that 98.3% of genes were expressed at comparable levels across N-, P-, and B_12_-lim, while only 1.7% (443 genes) were differentially expressed (|log₂FC| > 3, padj <0.05; Fig. 5a, Table S7). Differentially Expressed Genes (DEGs) were grouped into four clusters showing distinct functional enrichments (Fig. 5b), although most (64–80%) lacked annotation.

Cluster 1 comprised 90 genes upregulated under P-lim but downregulated under N- and B_12_-lim, enriched in intracellular trafficking, secretion, and vesicular transport, with several photosynthesis-related genes and genes for P transport/remobilization. Cluster 2 included 153 genes upregulated under P- and N-lim but repressed under B_12_-lim, enriched in posttranslational modification, protein turnover, and chaperone functions, including carbon metabolism and stress response genes. Cluster 3 consisted of 103 genes upregulated under B_12_-lim and moderately under P-lim, but downregulated under N-lim, dominated by genes of unknown function plus transcription/translation-related genes. Cluster 4 comprised 97 genes upregulated under B_12_-lim and moderately under N-lim, enriched in amino acid and nucleotide transport/metabolism, including multiple genes for N metabolism and transport (Fig. 5c).

Of particular interest, the B_12_-dependent methionine synthase gene *metH* was expressed in all three nutrient limitation conditions without significant differences in expression suggesting an obligate dependency on B_12_ (Table S8).

To explore the influence of the SynCom on *P. parvum* under B_12_-lim, transcriptomes with and without SynCom were compared on Day 14. Most genes (98.8%) were expressed at comparable levels with or without the SynCom, while 1.2% were condition-specific, corresponding to 297 DEGs (|log₂FC| > 3, padj <0.05; Fig. 5d, Table S9) grouped into two clusters (Fig. 5e). Cluster 1 (273 genes) was upregulated without the SynCom, enriched in posttranslational modification, protein turnover, and chaperones, suggesting a stress-related state. Cluster 2 (24 genes) was upregulated with the SynCom and repressed without, including genes involved in amino acid transport/metabolism, transcription, translation, replication, and unknown functions, signature of growth reactivation (Fig. 5f).

The *metH* gene was expressed at similar levels in both conditions also confirming *P. parvum* B_12_ dependency (Table S8).

#### SynCom transcriptional responses

**Figure 6:**
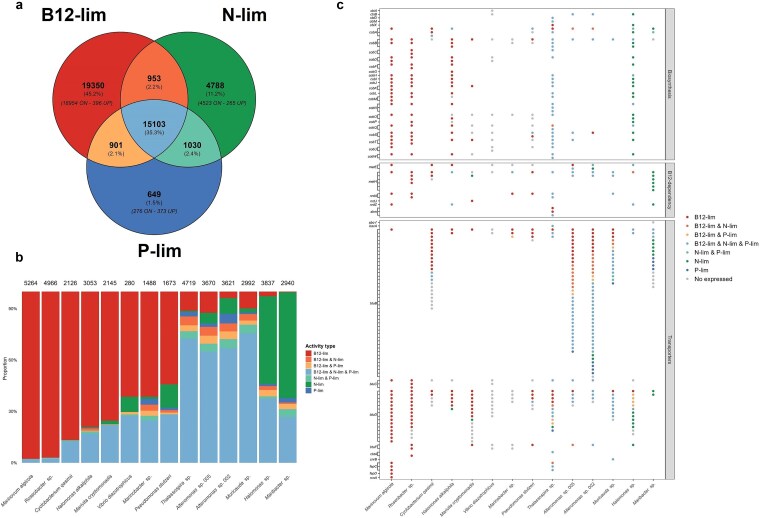
Transcriptional profiles of the SynCom under nutrient-limited conditions. **(a)** Venn diagram summarizing bacterial gene expression in *P. parvum* co-cultures under N-, P-, and B_12_-lim at Day 14. Values in bold in each section indicate the total number of genes expressed and upregulated in the corresponding condition. Numbers below further distinguish genes that were upregulated (“UP”) from those expressed exclusively in a given condition (“ON”). **(b)** Distribution of expressed genes across SynCom bacterial strains. Barplots show the proportion of expressed genes for each of the 14 SynCom strains detected by metatranscriptomics (with *V. aestuarianus* excluded as it was not detected). Numbers above bars indicate the total number of expressed genes per strain. Colors represent the nutrient-limitation conditions in which genes were expressed: single limitations (N-, P-, or B_12_-lim), pairwise combinations (N + P, N + B_12_, P + B_12_), or all three limitations (N + P + B_12_). **(c)** Expression patterns of targeted SynCom genes involved in vitamin B_12_ metabolism across nutrient-limiting conditions. The x-axis lists SynCom bacterial strains and the y-axis shows selected genes grouped into three functional categories: B_12_ biosynthesis genes, B_12_-dependent genes (including *metE* for comparison with *metH* expression), and known B_12_ transporter genes. Gene expression patterns are indicated by color.

A Venn diagram combining gene presence/absence and differential expression showed that 35.3% of bacterial genes were expressed across all nutrient conditions, 6.7% were shared between at least two conditions, and the remainder were condition-specific: 45.2% B_12_-lim, 11.2% N-lim, 1.5% P-lim (Fig. 6a). Expression varied among SynCom members: *Vibrio aestuarianus* showed no detectable expression, *V. diazotrophicus* a limited activity (280 genes), while other strains expressed 1488 to 5264 genes (Fig. 6b).

Bacterial strains displayed four distinct patterns of expression. “Highly B_12_-lim-specific strains”, such as *Marinovum algicola* and *Roseobacter* sp., had over 90% of their genes uniquely expressed or upregulated under B_12_-lim. Six “moderatly B_12_-lim-specific strains”, including *Cyclobacterium qasimii* and *Halomonas alkaliphila*, had more than 50% of genes uniquely expressed or upregulated under B_12_-lim. “Generalist strains”, including *Thalassospira* sp., the two *Alteromonas* strains, and *Muricauda* sp., expressed over 60% of their genes across all three nutrient limitation conditions. Finally, “moderatly N-lim-specific strains”, such as *Halomonas* sp. and *Maribacter* sp., had more than 50% of genes uniquely expressed or upregulated only under N-lim (Fig. 6b). These strategies suggest complementary roles that maintain holobiont stability under fluctuating nutrient limitation.

Clusters of Orthologous Groups of proteins enrichment analysis of the generalist bacteria revealed shared metabolic signatures across nutrient limitations (Fig. S7). Under P-lim, *Alteromonas* sp. 002, *Muricauda* sp., and *Thalassospira* sp. increased functional potential related to inorganic ion transport and metabolism. Both *Alteromonas* strains upregulated genes associated to cell motility under N- and P-limitation, while transcription, replication, lipid metabolism, vesicular transport, and carbohydrate metabolism were predominantly upregulated under B_12_-lim by *Alteromonas* sp. 005, *Muricauda* sp., and *Thalassospira* sp. These patterns indicate that generalists provide flexible metabolic capacity that buffers the holobiont.

#### Focus on B_12_ genes-related expression

Given that *P. parvum* resumed growth under B_12_-lim from Day 9 only in the presence of the SynCom, we examined bacterial expression of vitamin B_12_-related genes across SynCom members to elucidate the contribution of each bacterial strain (Fig. 6c). Five strains, *Marinovum algicola, Roseobacter* sp., *Halomonas alkaliphila, Halomonas* sp., and *Thalassospira* sp. possessed the complete B_12_ biosynthetic pathway. Among these, *Marinovum algicola, Roseobacte*r sp., and *Halomonas alkaliphila* exhibited a “B_12_-lim-specific” profile, with most genes, including those of the aerobic B_12_ biosynthetic pathway, expressed exclusively under B_12_-limited conditions, suggesting B_12_ production. *Thalassospira* sp. displayed a “generalist” pattern with broad expression of its B_12_ biosynthetic genes across all nutrient limitation conditions. Only three genes showed condition-specific regulation: *cbiM*, an essential component of the aerobic cobalamin biosynthetic pathway, and *cbiX*, whose annotation (the “X” denoting unknown function) prevents precise interpretation beyond its general implication in the pathway, were significantly upregulated under B_12_-lim. In addition, *cobQ*, which expression has been correlated to B_12_ production [56], was significantly upregulated under both B_12_- and N-lim. However, *cobQ* is not strictly essential, since its function can be complemented by *cbiP*, which catalyzes the same step in the pathway. *Halomonas* sp. showed a “N-lim-specific” profile, with most B_12_ biosynthetic genes upregulated under N-lim.

To assess B_12_ dependency within the SynCom, we analyzed the presence and expression of key B_12_-dependent genes. All 14 transcriptionally active strains possessed *metH*, encoding the B_12_-dependent methionine synthase, while nine strains also carried the B_12_-independent *metE*. Several strains contained additional B_12_-dependent genes (*nrdA, nrdJ, nrdZ, sbm*) (Fig. 6c). Among non-B_12_ producers, *Cyclobacterium qasimii, Marivita cryptomonadis, Marinobacter* sp., and *Pseudomonas stutzeri* upregulated B_12_-dependent genes under B_12_-lim. Strains with both *metH* and *metE* showed differential regulation: *Marinovum algicola* and *Cyclobacterium qasimii* upregulated both, *Halomonas* sp., and *Pseudomonas stutzeri* upregulated *metH*, whereas *Halomonas alkaliphila* and *Alteromonas* sp. 005 upregulated *metE* under B_12_-lim.

Finally, to investigate B_12_ potential transport, we identified known cobalamin transport genes across seven transporter families, including the canonical btu system (*btuB, btuC, btuD, btuF*; [57]; Fig. 6c), implicated in B_12_ import. For the other transporter families, it remains unclear whether they mediate import or export of cobalamin. All bacterial members except *Vibrio diazotrophicus* expressed B_12_ transporters under B_12_-lim, with the number of homologues varying among strains. Notably, the non-B_12_ producers *Cyclobacterium qasimii, Marivita cryptomonadis, Marinobacter* sp., and *Pseudomonas stutzeri*, which also upregulated B_12_-dependent genes, strongly induced most of their B_12_ transporters under B_12_-lim conditions. This widespread induction suggests active competition for extracellular B_12_ within the holobiont, coupled with efficient acquisition by dependent taxa.

## Discussion

By integrating phenotypic measurements with a multi-omics approach, this study provides new insights into *P. parvum*-bacteria interactions within the algal holobiont under contrasting nutrient limitations. We establish a link between algal physiology and bacterial strategies, revealing bidirectional interactions in which nutrient availability drives reciprocal modulation of algal and bacterial metabolism, highlighting the dynamic nature of the *P. parvum* holobiont under nutrient limitation.

### Mutualistic interactions within the holobiont are driven by nutrient limitations

Under N- and P-lim, co-culturing *P. parvum* with the SynCom did not significantly affect algal growth while under B_12_-lim, a strong mutualistic interaction emerged: algal growth increased more than three-fold in the presence of the SynCom, while bacterial abundances were also higher than under N- or P-lim. These reciprocal benefits are consistent with algal vitamin auxotrophy and bacterial provision of B_12_, supported by algal-derived resources.

Previous studies have shown that nutrient limitation can modulate algal-bacterial interactions by reshaping SynCom dynamics [58], and that associated bacteria can promote algal growth through vitamins and phytohormones [27]. Previous work further emphasized that metabolic context, particularly auxotrophy and nutrient stress, determines whether interactions result in mutualism, neutrality, or competition [59, 60]. Building on these findings, our study demonstrates that within our SynCom, vitamin B_12_ limitation constitutes the key context triggering strong reciprocal interactions with *P. parvum*, reinforcing the idea that metabolic dependencies such as vitamin auxotrophy are decisive drivers of holobiont mutualism [18, 61]. A recent study further supports the broader relevance of B_12_, showing that it is a key factor shaping prokaryotic community clustering across seasons, highlighting potential implications for phytoplankton bloom dynamics beyond *P. parvum* [62].

The absence of growth stimulation under N- or P-lim may reflect insufficient bacterial nutrient cycling or preferential retention of regenerated nutrients by bacteria. Although associated bacteria can recycle nutrients and supply vitamins such as B_12_ [63], such support may be insufficient or mismatched to algal demand. Preferential nutrient retention can further reduce availability to phytoplankton [64], and mutualistic interaction through N remineralization strongly depends on the physiological state of both partners [65]. In addition, for N fixation, the oxygen sensitivity of the nitrogenase complex may have constrained activity in our oxic batch cultures [66], suggesting that microscale organization [67] or protective respiration [68] may be required for non-cyanobacterial diazotrophs to sustain algal growth under aerobic conditions.

### Prymnesin production is induced by macronutrient stress

Beyond growth dynamics, nutrient limitation profoundly shapes specialized metabolism, notably toxin production. P stress has previously been associated with enhanced toxicity in *P. parvum*, potentially reflecting a reallocation of cellular resources toward secondary metabolism when growth is constrained [38, 69] and with ecological consequences documented in natural blooms [70]. While earlier studies relied on indirect measurements, mass spectrometry analyses have confirmed that P limitation enhances PRM production [46].

Here, we provide the first direct comparison of PRM production under N, P, and vitamin B_12_ limitation during the early stationary phase of *P. parvum* in each treatment, as determined from growth curves. Our results show that PRM accumulation is specifically modulated by macronutrient stress rather than by micronutrient limitation, refining previous notions that nutrient stress broadly triggers toxin production [36, 71]. One explanation is that N- and P-lim generate excess fixed carbon that can be redirected toward secondary metabolism, whereas B_12_-lim primarily impairs cofactor-dependent enzymatic reactions and slows overall metabolism [72], preventing carbon accumulation and toxin biosynthesis.

The presence of the SynCom did not significantly affect PRM levels under any nutrient condition, consistent with previous observations [39], but contrasting with other systems such as *Alexandrium pacificum* co-cultured with *Jannaschia cystaugens*, where bacterial presence significantly increases toxin production [73]. These results indicate that, under our experimental conditions, PRM biosynthesis in *P. parvum* was primarily associated with macronutrient stress rather than micronutrient stress or the presence of the SynCom. However, we cannot exclude that specific bacterial signals, different incubation times, or alternative bacteria-to-alga ratios may influence prymnesin production. Dedicated experiments would be required to directly test the effect of bacterial exometabolites or individual strains on toxin biosynthesis.

### A potential holobiont metabolic signature of *P. parvum* resumed growth

Nutrient limitation reshaped both the endo- and exometabolomes of *P. parvum*, with a clear separation of N-, P-, and B_12_-lim conditions. Comparable stress-driven metabolic shifts have been reported in other algal groups [74]. Under B_12_-lim, metabolomic profiles further distinguished cultures with and without the SynCom, consistent with previous reports showing that microbial partners influence algal metabolic landscapes [60, 75, 76]. These findings highlight that metabolomic outputs integrate both endogenous adjustments to nutrient stress and exogenous modifications mediated by microbial associations.

Across all nutrient limitations, sulfobacin-like compounds were consistently enriched in co-cultures, suggesting a bacterial origin. Although sulfonolipids such as sulfobacins have not previously been described in algal-bacterial systems, their structural similarity to bioactive sulfonamides which exhibit antibacterial activity [77, 78], raises the possibility that they could contribute to microbial interactions or pathogen defense.

Under B_12_-lim, co-cultures showed a distinct metabolic profile characterized by ectoine accumulation, consistent with enhanced osmoprotection [79]. Ectoine, produced primarily by halophilic bacteria such as *Halomonas* spp., and also reported in *P. parvum* [79, 80], protects cells against multiple stressors [81] and may reflect a joint survival strategy enhancing holobiont resilience. In parallel, increases in membrane lipids suggest membrane-remodeling [82] preceding growth recovery. The function of the strongly accumulated unknown metabolite M251T499 remains unclear. Some metabolites enriched in co-culture, including ectoine and PC/PE lipids, may also provide carbon and N substrates for bacteria [83, 84]. Conversely, thiamine-like compounds were strongly reduced, consistent with cofactor reallocation under B_12_-lim [85]. Decreases in glycerolipids, glycosylated metabolites, and DGCC-like lipids further suggest attenuation of stress-related lipid pools [86]. Although cultures remained in plateau phase at Day 8, these metabolic shifts anticipated the growth recovery observed at Day 10, indicating that the SynCom facilitated a transition toward renewed algal proliferation.

### Transcriptional reprogramming of *P. parvum* under nutrient limitation

Transcriptomic profiling revealed nutrient-specific transcriptional responses in *P. parvum*. Under P-lim, responses appear to extend beyond P acquisition to include vesicular trafficking and photosyn-thesis-related functions, suggesting broad cellular reorganization to optimize resource use. N-lim was characterized by induction of stress-related genes alongside N transporters and metabolic enzymes, indicating concurrent activation of coping mechanisms and assimilation pathways. In both conditions, stress-responsive transcripts predominated, consistent with the possibility of a generalized stress program involving protein turnover, chaperone activity, and metabolic downscaling as previously described [71, 87]. By comparing cultures at the same physiological state under well-defined N, P, and B_12_-limitation, our study isolates nutrient-specific responses while minimizing growth-stage effects.

In contrast, under B_12_-lim, the presence of the SynCom induced a distinct trajectory, with a shift toward growth-associated gene expression, including transcription, translation, amino acid metabolism, and N transport. This pattern indicates that bacterial B_12_ provisioning enabled *P. parvum* to resume metabolic activity, with N emerging as the next limiting resource. Similar vitamin-mediated recoveries have been documented in other algal-bacterial systems [18, 22, 61], underscoring the general importance of vitamin exchange in shaping algal physiology.

### B_12_ fluxes within *P. parvum* holobiont

Metatranscriptomic profiling of the SynCom revealed functional specialization underlying holobiont-level responses. Although community composition remained broadly stable, with only a slight enrichment of B_12_ producers under B_12_-lim, metatranscriptomics revealed contrasting bacterial strategies, with condition-specific specialists (e.g. B_12_ producers under B_12_-lim) and broadly responsive generalists contributing to community function, consistent with recent ecological frameworks [88, 89]. Enrichment of B_12_-producing taxa (i.e. *Marinovum algicola, Roseobacter sp., Halomonas sp.*) under B_12_-lim coincided with the upregulation of cobalamin biosynthesis pathways, enabling algal growth recovery and induction of growth-associated algal genes. Similar bacterial B_12_ provisioning has been shown to restore algal metabolism in other systems [61, 90, 91], although such exchanges are not universal [22]. Together, metabarcoding and metatranscriptomics show that SynCom resilience stems not only from membership but also from functional reprogramming under nutrient stress.

**Figure 7:**
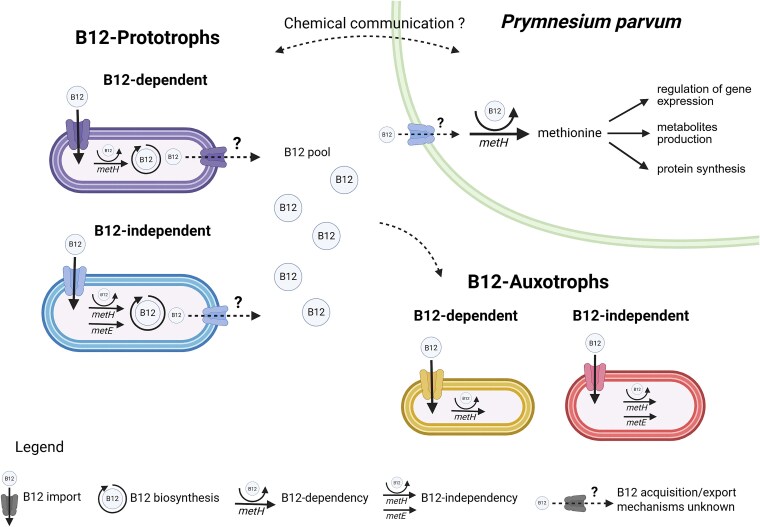
Conceptual model of vitamin B_12_-mediated interactions within the algal-bacterial holobiont. Bacterial strains are classified according to the expression of genes involved in vitamin B_12_ biosynthesis and B_12_ dependence based on methionine synthesis pathways. B_12_-dependent bacteria rely exclusively on the B_12_-dependent methionine synthase (*metH*), whereas B_12_-independent bacteria express both *metE* and *metH*. B_12_-prototrophs express the cobalamin biosynthetic pathway, while B_12_-auxotrophs lack its expression and rely on external sources of vitamin B_12_. All bacterial groups are able to import vitamin B_12_, while B_12_ release by producers is hypothesized and remains unresolved. Vitamin B_12_ exchanged within the holobiont supports methionine synthesis in the B_12_-dependent microalga *P. parvum*, thereby sustaining cellular growth and metabolism. Dashed arrows indicate hypothesized interactions. Created in BioRender. Réveillon, D. (2026) https://BioRender.com/om58389

SynCom bacteria displayed complementary B_12_-related strategies along three axes: production, dependence, and import (Fig. 7). B_12_-prototrophs expressed the complete biosynthetic pathway, whereas auxotrophs relied on external sources. Facultatively B_12_-dependent taxa expressed both *metH* (B_12_-dependent) and *metE* (B_12_-independent), allowing methionine synthesis regardless of vitamin availability, while strictly B_12_-dependent taxa expressed only *metH*. Notably, B_12_ independence does not preclude auxotrophy, as several non-producing taxa expressed *metE* while lacking the biosynthetic pathway. All members expressed B_12_ transporters, which were strongly upregulated under B_12_ limitation alongside B_12_-dependent enzymes, reflecting active acquisition and reinforcing functional complementarity. Recent work has shown that bacteria auxotrophic for the complete B_12_ biosynthesis pathway, but capable of producing individual building blocks, can jointly synthesize [92]. Some strains in our SynCom are capable of completing the final steps of B_12_ biosynthesis, suggesting that similar joint prototrophy could theoretically occur within this community, although these genes were not strongly upregulated under B_12_-lim in our experiments. Such interactions could add an additional layer of complexity to vitamin-mediated microbial cooperation.

The genomic features of *P. parvum* further shape this exchange. Like other algae that have evolved in close association with B_12_-producing bacteria [93], *P. parvum* lacks the B_12_-independent methionine synthase *metE* and relies exclusively on the B_12_-dependent isoform *metH*. Consistent with this obligate auxotrophy, *metH* was robustly expressed across all conditions and did not differ between cultures with or without the SynCom. This constitutive expression aligns with observations that microalgae do not modulate *metH* expression in response to intracellular B_12_ availability [94], maintaining the methionine cycle poised to exploit any available vitamin. The lack of variation in *metH* expression across treatments suggests that transcription of the B_12_-dependent methionine synthase is not directly responsive to external vitamin B_12_ availability. Instead, the observed growth differences under B_12_-lim likely reflect variation in actual vitamin acquisition and intracellular availability, which are not directly captured by *metH* expression levels.

Together, these results suggest a holobiont-level mutualism. Heterotrophic bacteria dependent on algal organic matter synthesize and mobilize B_12_ under nutrient stress, and both partners utilize it. Such reciprocal adjustments exemplify mutualistic interactions consistent with models of symbiotic vitamin exchange in natural phytoplankton-associated communities [95].

The precise mechanism of B_12_ transfer remains unresolved. Not only are the potential acquisition routes unclear, but the identity of bacterial exporters mediating vitamin release is also unknown. Vitamin B_12_ can be acquired either actively, through bacterivory and direct access to intracellular bacterial pools [96], or passively, via uptake of dissolved vitamin released into the environment by leakage [97], cell lysis [98], or virus-driven processes, including prophage induction [92], and infection by exogenous lytic phages, as shown in *Sulfitobacter* sp. M39 [99]. Several possibilities are plausible, including uptake of extracellular vitamin passively released by bacteria, bacterivory access to intracellular pools following bacterial lysis release triggered by prophage induction or even phage-mediated release, as recently shown in *Sulfitobacter* sp. M39. Although extracellular B_12_ was not detected, likely due to methodological constraints and low concentrations, the strong upregulation of bacterial importers and B_12_-dependent enzymes indicates that vitamin was available and actively utilized. These observations strongly suggest that passive release by producers and active uptake by dependent partners are central components of B_12_ exchange within the holobiont.

## Conclusion

This study shows that nutrient availability, particularly vitamin B_12_, is associated with marked physiological, metabolic, and transcriptional changes in the *P. parvum* holobiont under our experimental conditions. Taken together, our results support the idea that vitamin B_12_ limitation can modulate phytoplankton-bacteria interactions during bloom-like scenarios, although the magnitude and ecological significance of these effects likely depend on environmental context. While our simplified SynCom does not capture the diversity and complexity of natural microbial assemblages, it provides a controlled framework to explore metabolic complementarity between algae and bacteria. Our findings therefore suggest that vitamin-mediated interactions could contribute to regulating algal growth dynamics under certain conditions. However, extrapolation to natural systems should be made with caution, as bloom magnitude, persistence, and toxicity are shaped by multiple interacting biotic and abiotic factors. Further studies in more complex and environmentally realistic systems will be necessary to determine the broader ecological implications for food web dynamics, harmful algal bloom development, fisheries, and aquaculture under changing nutrient regimes.

## Supplementary Material

Supplementary_material_ycag142

## Data Availability

The datasets generated and analysed during the current study are available in the following repositories: Metabarcoding, RNA-seq and bacterial genome sequencing data are available in the European Nucleotide Archive under project accession PRJEB106332 and are accessible at this repository Metabolomics datasets are available at this repository The *P. parvum* genome assembly is available at this repository The code used for data analysis and figure generation is available on GitLab.
