# Supplementary material for: Multi-omics uncovers nutrient stress-driven interactions in a synthetic Prymnesium parvum holobiont, with vitamin B12-limitation revealing mutualism
Source: ISME Commun. 2026 May 22;6(1):ycag142. doi: 10.1093/ismeco/ycag142 (PMC13431280; doi:10.1093/ismeco/ycag142)
Supplement: Supplementary_material_ycag142 [file supplementary_material_ycag142.zip › Supplementary data_Patron_et_al.docx]

*
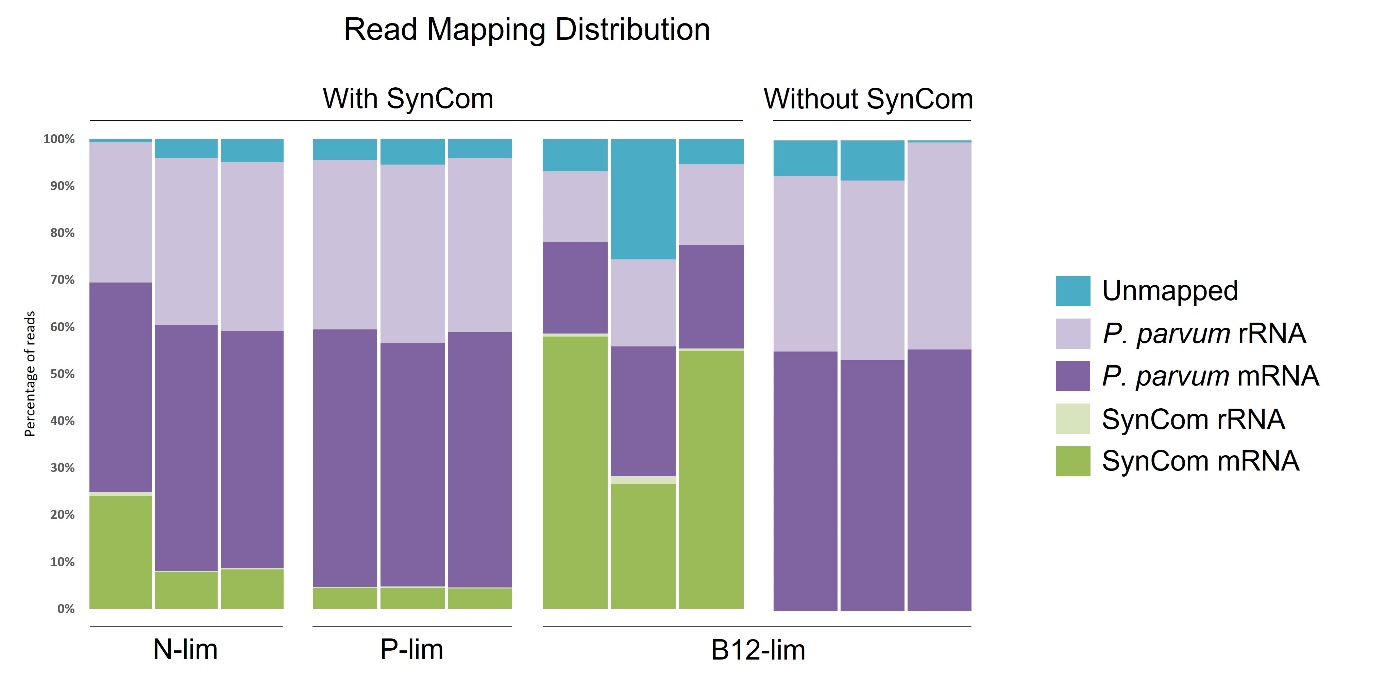
*

# Supplementary Figure S1. Distribution of transcriptomic reads across categories under N-, P-, and B12-lim on day 14.

Each sample generated 30-40 million reads. In SynCom-containing cultures, read distribution varied according to nutrient condition: *P. parvum* mRNA accounted for 42 ± 15% of reads, *P. parvum* rRNA for 29 ± 10%, SynCom rRNA for 1 ± 1%, and unmapped reads for 6 ± 8%. SynCom mRNA abundance differed markedly across conditions, representing 47 ± 17% of total reads under B12-lim but only 9 ± 8% under N- and P-lim. In *P. parvum* monocultures, reads mapped predominantly to *P. parvum*: 55 ± 1% to mRNA and 40 ± 4% to rRNA, with unmapped reads contributing 6 ± 4% of the total.

*
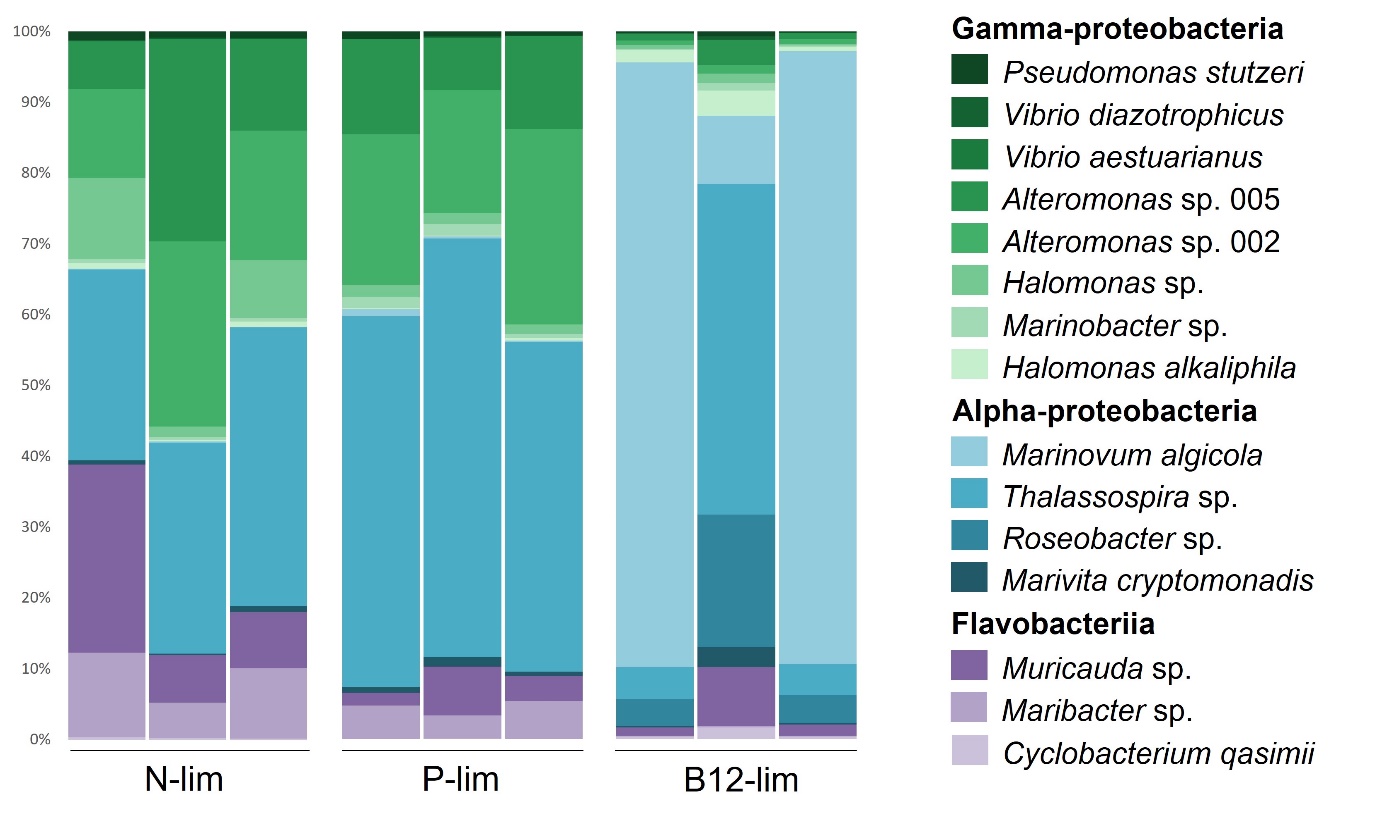
*

# Supplementary Figure S2. Taxonomic composition of SynCom mRNA reads under N-, P-, and B12-limitation on day 14.

Under N- and P-lim, *Thalassospira* sp. dominated the SynCom transcriptome (42% ± 13% of reads), with the two *Alteromonas* strains contributing 34% ± 13%. In B12-lim, *Marinovum algicola* and *Roseobacter* sp. became dominant (61% ± 44% and 9% ± 9%), while other strains, including the two *Vibrio* spp., *Cyclobacterium qasimii*, and *Marinobacter* sp., each contributed less than 2%.

#
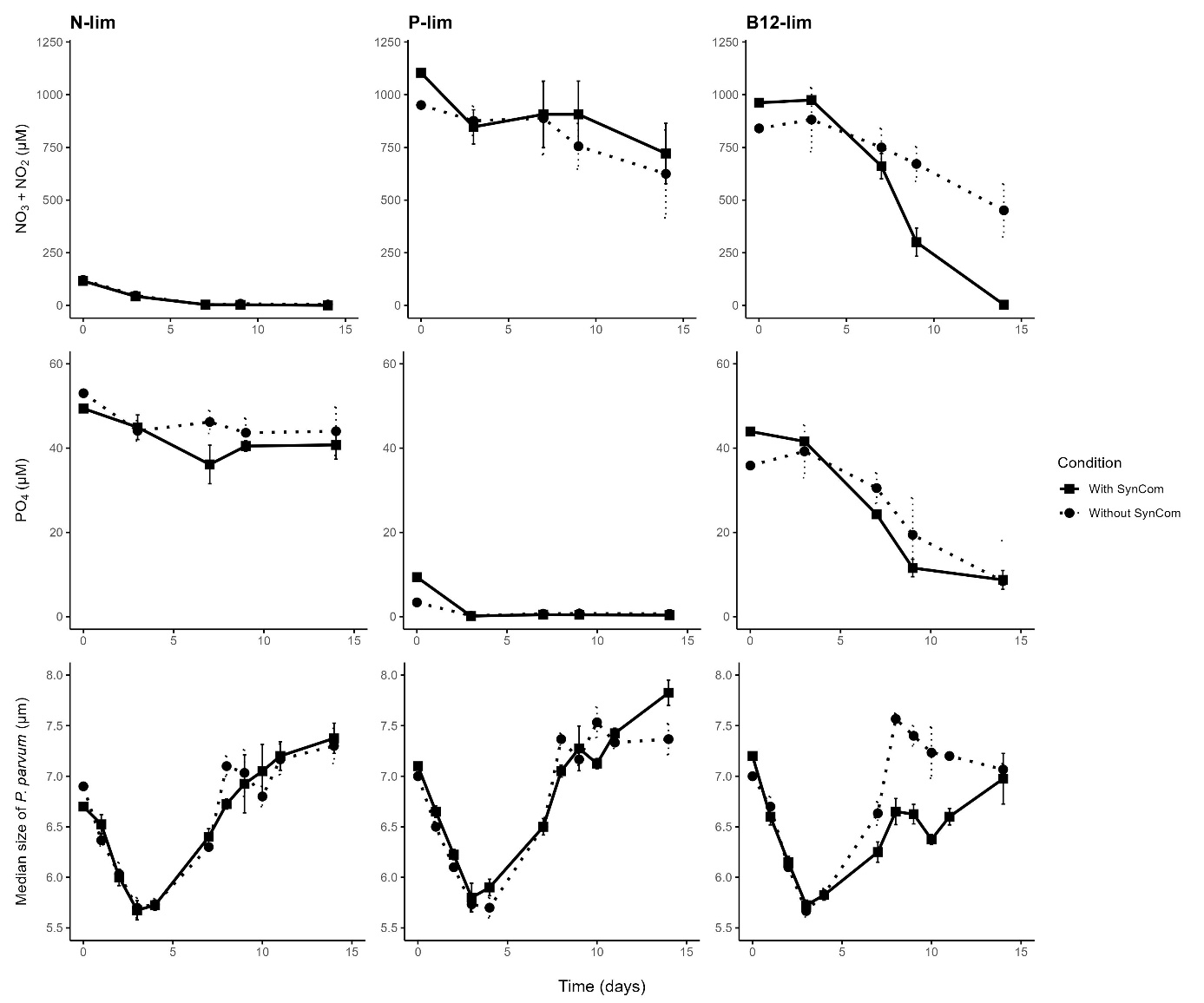
Supplementary Figure S3. Individual growth curves of P. parvum with and without SynCom.

(A) Growth curves of *P. parvum* in the presence of the SynCom. Each plot shows cell concentration (cells/mL) over time for one biological replicate. Plots are arranged in a 3 × 4 grid: rows indicate nutrient limitation (Row 1: N-lim, Row 2: P-lim, Row 3: B12-lim), and columns indicate spike type applied at day 14 (Column 1: N spike, Column 2: P spike, Column 3: B12 spike, Column 4: Seawater control). Each subplot corresponds to one replicate (n = 4 per condition), labeled at the top (“Replicate 1”, etc.). The red dashed vertical line indicates the spike day (day 14). (B) Growth curves of *P. parvum* in the absence of the SynCom. Plots are arranged in a 3 × 3 grid (one replicate missing), with rows for nutrient limitation (N-lim, P-lim, B12-lim) and columns for spike type (N, P, B12).


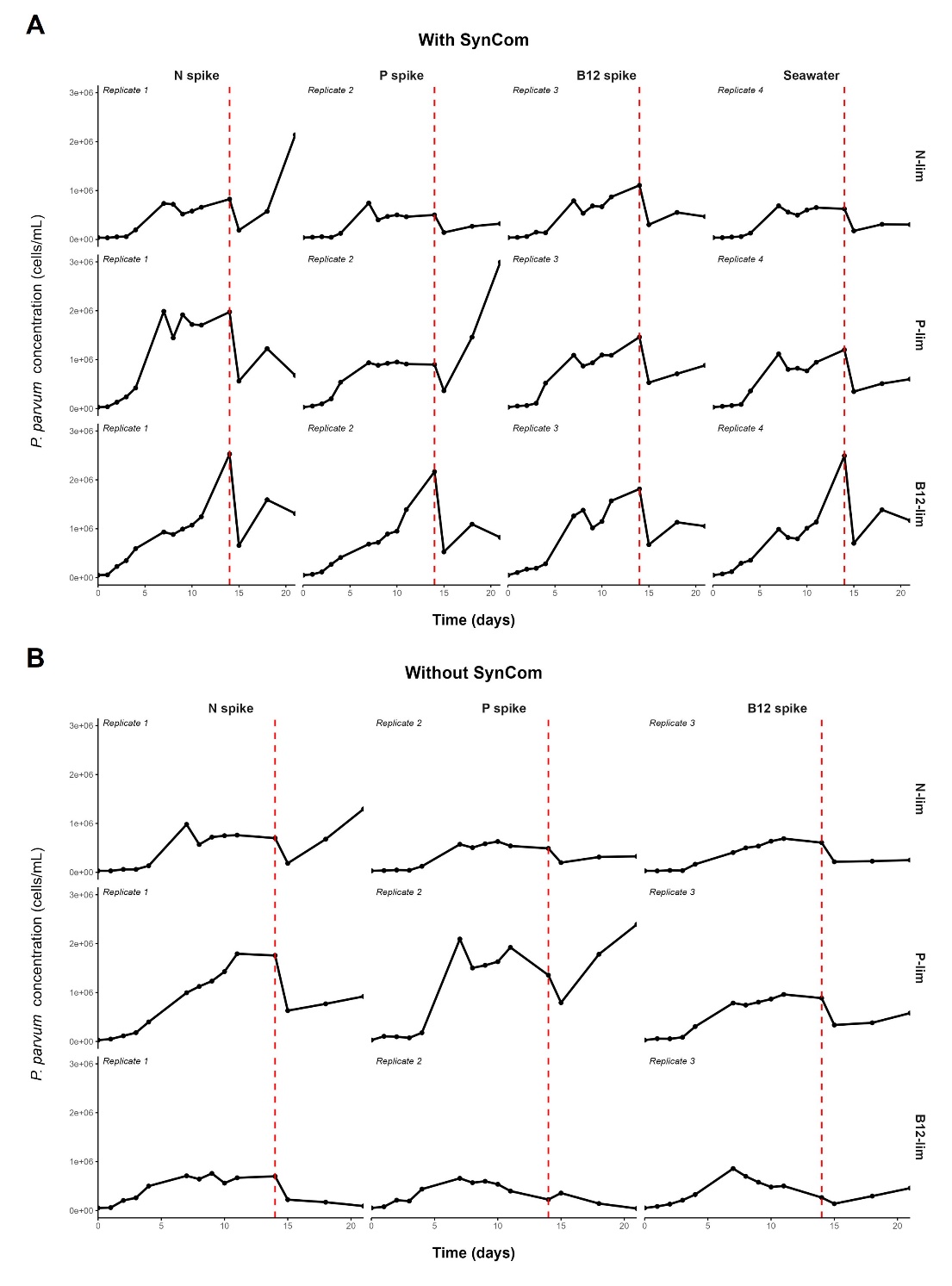


# Supplementary Figure S4. Temporal dynamics of physiological and environmental parameters under nutrient-limited conditions.

Each column represents a nutrient limitation regime: N-lim; P-lim and B12-lim cultures. From top to bottom, the panels show: Dissolved inorganic nitrogen (NO₃⁻ + NO₂⁻, in µM), Dissolved phosphate (PO₄³⁻, in µM), and median cell size of *P. parvum* (in µm). Data are plotted over time. Solid lines correspond to the With SynCom condition, and dashed lines represent the Without SynCom condition. The median cell size was acquired using a Multisizer 4 Coulter Counter (Beckman Coulter, Indiana, USA).


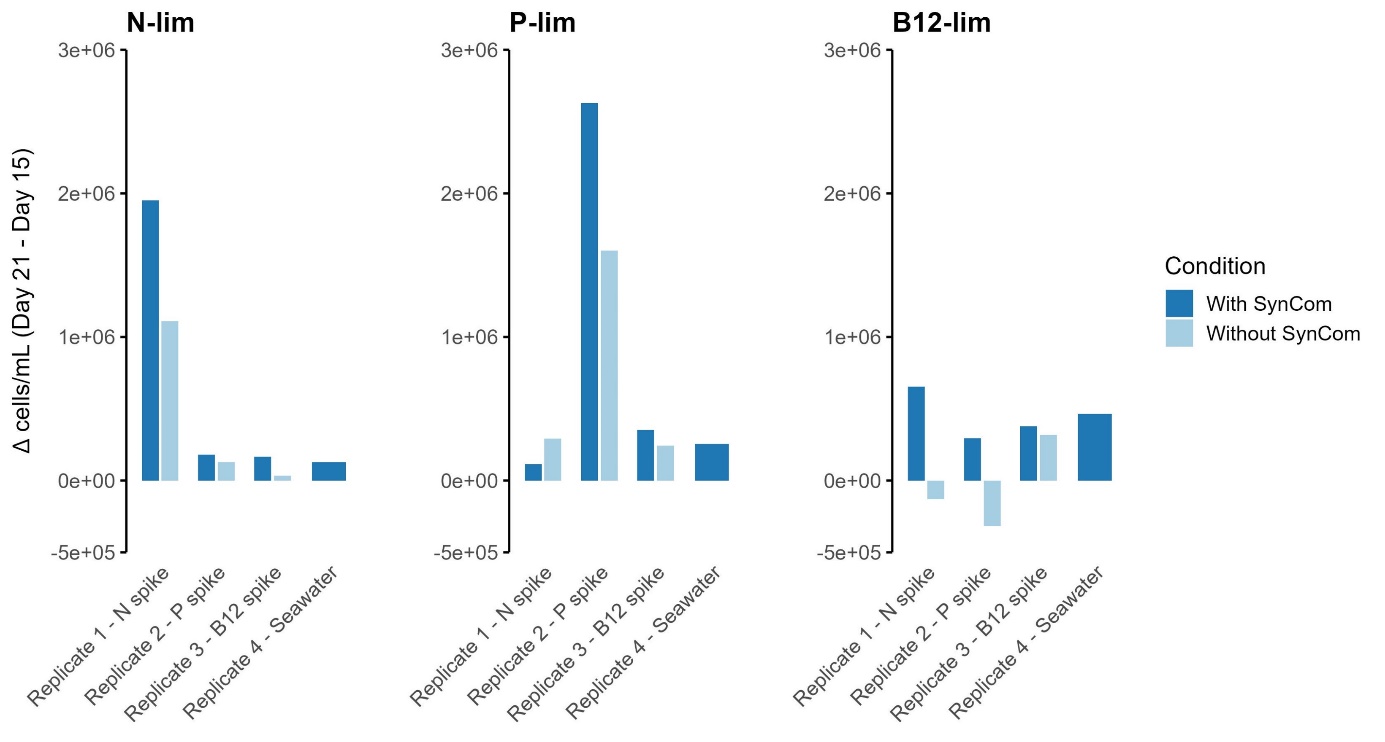


# Supplementary Figure S5. Effect of nutrient spikes on P. parvum growth recovery.

Barplots show the change in algal cell concentration (Δ cells/mL between day 21 and day 15) under three limitation regimes: (A) N-lim, (B) P-lim, and (C) B12-lim cultures. Each plot displays four replicates corresponding to different nutrient spikes (N, P, B12, or seawater control) as well as the presence or not of the SynCom.

In N- and P-lim conditions, only the addition of the corresponding nutrient restored growth. Under B12-lim, all spikes induced moderate growth increases in the presence of SynCom, whereas without SynCom only B12 addition produced a response. Depletion of dissolved N and P (Figure S2), as well as elevated C/N ratios in N-lim cultures (Figure S4), further validated the limitation regimes.

#
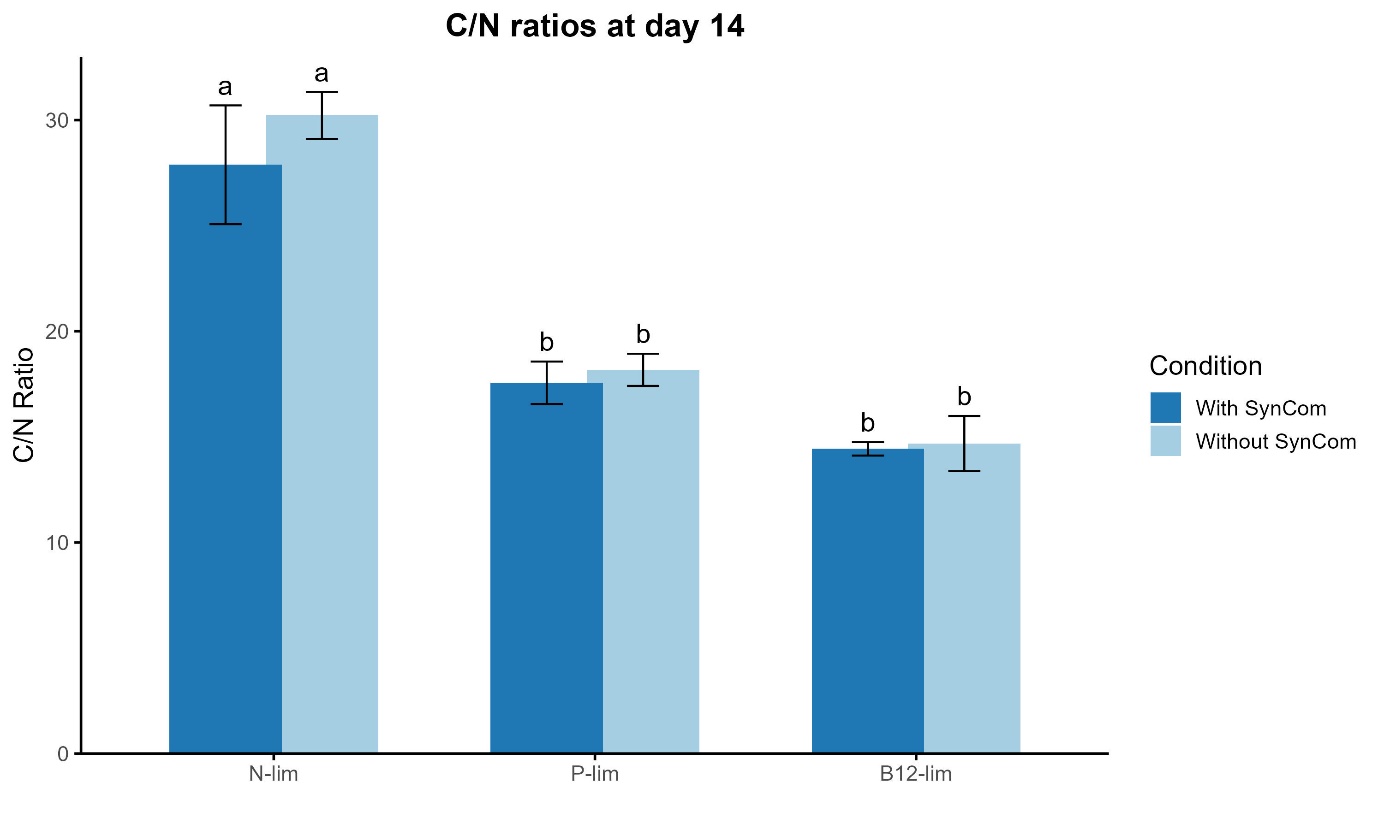
Supplementary Figure S6. C/N ratios of P. parvum at day 14 under nutrient limitation.

The barplot shows C/N ratios measured in cultures limited in N-, P- and B12-lim conditions at day 14 with or without the SynCom.

#
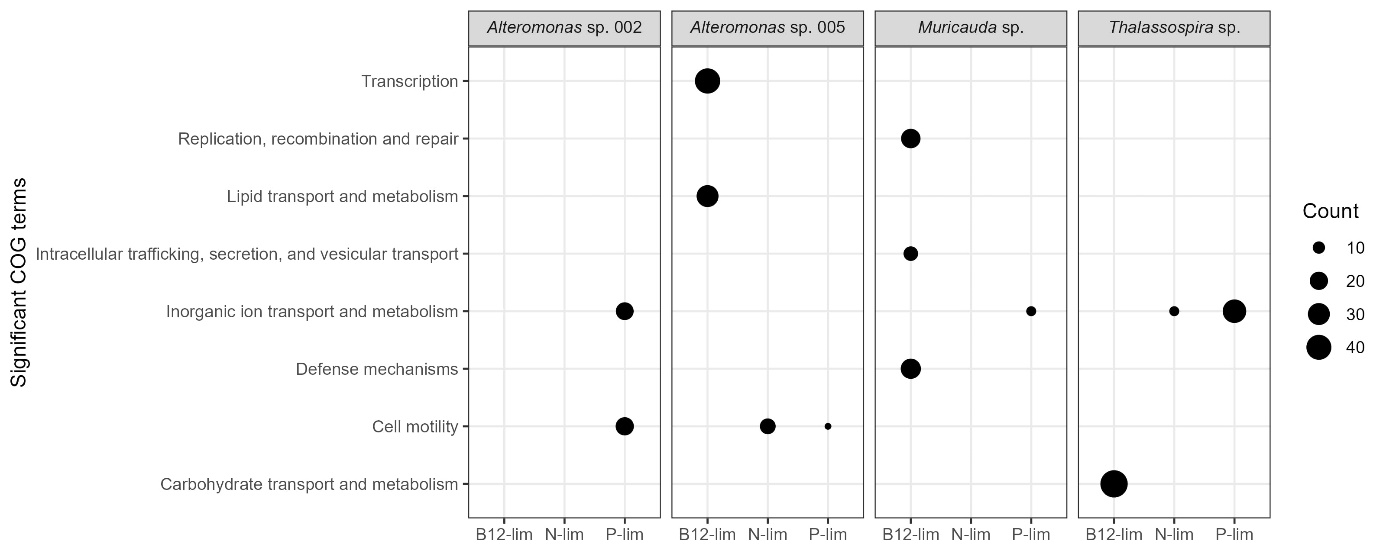
Supplementary Figure S7. Functional enrichment analysis of the four plastic-responsive bacterial strains.

Each panel corresponds to one of the four plastic-responsive bacterial strains. Within each panel, the X-axis shows the nutrient-limitation conditions (B12-, N- and P-lim), and the Y-axis lists the significantly enriched COG functional categories. Circles represent COG categories in which genes were significantly overexpressed under the corresponding nutrient-limitation condition; circle size is proportional to the number of overexpressed genes in that category.

# Supplementary Table S1. Composition of the SynCom, origin, access to the genomes and strains.

# Supplementary Table S2. Targeted quantification of PRM1 and PRM2 in intracellular and extracellular fractions.

This table reports the targeted LC-MS quantification of PRM1 and PRM2 in intracellular and extracellular samples collected from cultures grown with or without the SynCom under N-, P-, and B12-lim. For chromatogram extraction, four selected ions corresponding to each compound were used: [M + 2H]²⁺, [M + 2H – pentose]²⁺, [M + H + Na]²⁺, and [M + H + NH₄]²⁺. The sum of the extracted ion chromatograms (EICs) for these four ions was used to generate the analytical chromatogram, and the integrated peak area was considered as the analytical response. For each sample, the table provides cell concentration (cells mL⁻¹), sampled volume, recovery volume, calculated cell concentration in the extract, raw peak areas for PRM1 and PRM2, and normalized values expressed as area per cell. Mean values (µ) and standard deviations (s) are provided where applicable. Experimental conditions (with or without SynCom), nutrient limitation (N, P, or B), and the combined signal (PRM1 + PRM2) are also indicated.

# Supplementary Table S3. Metabolomics analysis of intracellular and extracellular metabolites significantly affected by the presence of the SynCom.

This table lists metabolites that were significantly differentially produced between conditions with and without the SynCom, for both intracellular and extracellular fractions. Each row corresponds to a metabolomic feature identified by its unique MXXTXX code. Reported parameters include fold change (FC), log2(FC), adjusted p-value (p.adjusted), -log10(p), and significance status, as well as mass-to-charge ratio (m/z), retention time (RT), adduct and isotope information, and MS/MS availability. Putative metabolite annotations were obtained using Flash Entropy, GNPS spectral library matching, and SIRIUS, including identity or cosine scores, shared peaks, molecular formulae, compound class predictions (CANOPUS), and annotation confidence levels according to the Schymanski et al. (2014) classification.

# Supplementary Table S4. Data matrix of intracellular samples used for principal component analysis.

# Supplementary Table S5. Data matrix of extracellular samples used for principal component analysis.

# Supplementary Table S6. Weighting factors used for normalization of intracellular samples based on cell abundance.

This table reports the weighting factors applied to intracellular samples to account for differences in cell abundance between biological replicates and experimental conditions. For each replicate, the table provides sample name, code, replicate number, sample type, nutrient limitation, SynCom condition, sampling day, measured cell concentration (cells mL⁻¹), and calculated total number of cells per sampled volume. Two weighting factors are presented: (i) a global weighting factor calculated relative to the maximum total cell number across all samples, and (ii) a condition-specific weighting factor calculated relative to the maximum cell number within each nutrient limitation.

# *Supplementary Table S7. Differential gene expression analysis of Prymnesium parvum in response to the syncom under nutrient limitation.*

This table reports the 443 DEGs identified in *P. parvum* grown with or without the syncom under N-, P-, and B12-lim on day 14. Each row corresponds to one DEG, and columns include the comparison performed, gene identifier (GeneID), base mean expression (baseMean), log2 fold change (log2FoldChange), adjusted p-value (padj), and regulation status. Functional annotation fields comprise orthology assignments (seed_ortholog, eggNOG_OGs), annotation confidence levels, COG functional categories, gene descriptions and preferred names, as well as Gene Ontology (GO), enzyme commission (EC), and KEGG annotations (KO, pathways, modules, reactions, and related classifications). Additional annotations include BRITE hierarchies, transporters (KEGG_TC), carbohydrate-active enzymes (CAZy), metabolic reactions (BiGG), protein domains (PFAMs), clustering information, and sequence data. Remarks are provided where relevant.

Supplementary Table S8. Differential expression analysis of the metH gene in Prymnesium parvum.
This table focuses on the expression patterns of the B12-dependant methionine synthase gene (*metH*) in *P. parvum*. Differential expression analyses include comparisons between nutrient-limitation conditions (N-, P-, and B12-lim) as well as comparisons between cultures grown with and without the syncom, under B12-lim. Reported values include gene identifier (GeneID), comparison performed, mean normalized expression (baseMean), log2 fold change (log2FoldChange), standard error of the log2 fold change (lfcSE), test statistic (stat), raw p-value (pvalue), and adjusted p-value (padj).

# **Supplementary Table S9. Differential gene expression analysis of** *Prymnesium parvum* **under vitamin B12 limitation in response to the syncom at day 14.**

This table reports the 297 differentially expressed genes (DEGs) identified in *P. parvum* grown under B12-lim with or without the SynCom at day 14. Each row corresponds to one DEG, with columns indicating the comparison performed, mean normalized expression (baseMean), log2 fold change (log2FoldChange), adjusted p-value (padj), regulation status, and clustering assignment. Functional annotations include orthology information (seed_ortholog, eggNOG_OGs), annotation confidence level, COG functional categories, gene descriptions and preferred names, as well as Gene Ontology (GO), enzyme commission (EC), and KEGG annotations (KO, pathways, modules, reactions, rclass, BRITE hierarchies, transporters, CAZy families, and BiGG reactions). Protein domain information (PFAMs) and sequence data are also provided.
